# Recent Prospects in the Inline Monitoring of Nanocomposites and Nanocoatings by Optical Technologies

**DOI:** 10.3390/nano6080150

**Published:** 2016-08-19

**Authors:** Elodie Bugnicourt, Timothy Kehoe, Marcos Latorre, Cristina Serrano, Séverine Philippe, Markus Schmid

**Affiliations:** 1 Innovació i Recerca Industrial i Sostenible (IRIS), Parc Mediterrani de la Tecnologia, Avda. Carl Friedrich Gauss 11, Castelldefels, Barcelona 08860, Spain; cserrano@iris.cat; 2 IRIS Advanced Engineering, NexusUCD, University College Dublin, Blocks 9 & 10 Belfield Office Park Belfield, Dublin D04 V2N9, Ireland; tkehoe@iris.cat (T.K.); sphilippe@iris.cat (S.P.); 3 Instituto Tecnológico del Embalaje, Transporte Y Logística, Parque Tecnológico, C/Albert Einstein, 1, Paterna, Valencia 46980, Spain; marcos.latorre@itene.com; 4 Fraunhofer Institute for Process Engineering and Packaging IVV, Giggenhauser Strasse 35, Freising 85354, Germany; markus.schmid@ivv.fraunhofer.de; 5 Chair of Food Packaging Technology, Technische Universität München, Weihenstephaner Steig 22, Freising 85354, Germany

**Keywords:** nanocoatings, nanocomposites, inline monitoring, dispersion, thickness, spectroscopy, ellipsometry, spectral reflectance

## Abstract

Nanostructured materials have emerged as a key research field in order to confer materials with unique or enhanced properties. The performance of nanocomposites depends on a number of parameters, but the suitable dispersion of nanoparticles remains the key in order to obtain the full nanocomposites’ potential in terms of, e.g., flame retardance, mechanical, barrier, thermal properties, etc. Likewise, the performance of nanocoatings to obtain, for example, tailored surface affinity with selected liquids (e.g., for self-cleaning ability or anti-fog properties), protective effects against flame propagation, ultra violet (UV) radiation or gas permeation, is highly dependent on the nanocoating’s thickness and homogeneity. In terms of recent advances in the monitoring of nanocomposites and nanocoatings, this review discusses commonly-used offline characterization approaches, as well as promising inline systems. All in all, having good control over both the dispersion and thickness of these materials would help with reaching optimal and consistent properties to allow nanocomposites to extend their use.

## 1. Introduction

Nanostructured materials have emerged as a key research field in order to confer materials with unique or enhanced properties. Polymer nanocomposites are defined as solids consisting of a mixture of two or more phase-separated materials, with one or more dispersed phase in the nanoscale, and a polymer matrix. The nanoparticles used are considered nanoscale when one, two or three of their external dimensions range from approximately 1–100 nm [1]. Nanocomposites can be processed by conventional wet and dry processing techniques; however, conditions need to be adjusted compared to their neat counterparts. The performance of nanocomposites depends on a number of parameters, but the suitable dispersion of nanoparticles remains the key in order to obtain the full potential of the nanocomposites in terms of, e.g., flame retardance [2], mechanical and barrier properties [3].

Polymer nanocomposites and nanoparticles can also be applied as nanocoatings by different technologies, such as electrospray. The resulting deposited nanoscale layers can provide specific surface behaviour to the substrate where they are applied. Nanocoatings allow antimicrobial [4], gas barrier [5,6,7], water vapour barrier [8], water repellence [9] or flame retardant [10] surface functionalization and, therefore, provide interesting prospects for solar panels [11], packaging [5] and automotive parts [12], among others. The performance of nanocoatings to obtain, for example, tailored surface affinity with selected liquids (e.g., for self-cleaning ability), protective effects against flame propagation, UV transmission or gas permeation, is highly dependent on the nanocoatings’ thickness and homogeneity [13].

In addition, as opposed to standard composites, nanocomposites and nanocoatings present the advantage of being potentially transparent, although the optical properties can be highly affected by the nanocomposite morphology.

The nanoparticles’ dispersion can be characterized by different states at the nano-, micro- and macroscopic scales. The nanoparticles’ dispersion is generally characterized offline through the use of electron microscopy [14,15], X-ray diffraction etc., whereas nanocoating thickness is commonly assessed by electron microscopy or profilometry [16]. As reported in this review, a number of efforts, generally based on the insertion of optical probes, have also been undertaken to continuously monitor the processing of nanocomposites in order to help optimise the dispersion of nanoparticles [17,18,19,20] and/or the thickness of nanocoatings, which are key to the resulting performance.

The goal of this review is to discuss the commonly used approaches, as well as recent advances and promising inline monitoring systems for nanocomposites and nanocoatings. All in all, having good control over both the dispersion and thickness of these materials would help with reaching optimal and consistent properties to allow extension of the use of nanocomposites, as illustrated here in terms of the compositions and features of particular relevance in the packaging, solar energy and automotive industry.

## 2. Nanoparticles Dispersion Monitoring

### 2.1. Background

Due to their high specific surface area and commonly opposed polarity to that of polymeric matrices, one of the major challenges encountered when processing nanocomposites is the multi-scale aggregation and agglomeration of nanoparticles [21]. This often translates into an insufficient dispersion and, therefore, the poor properties of nanocomposites in practice compared to the promised enhancements reported in the literature.

**Figure 1:**
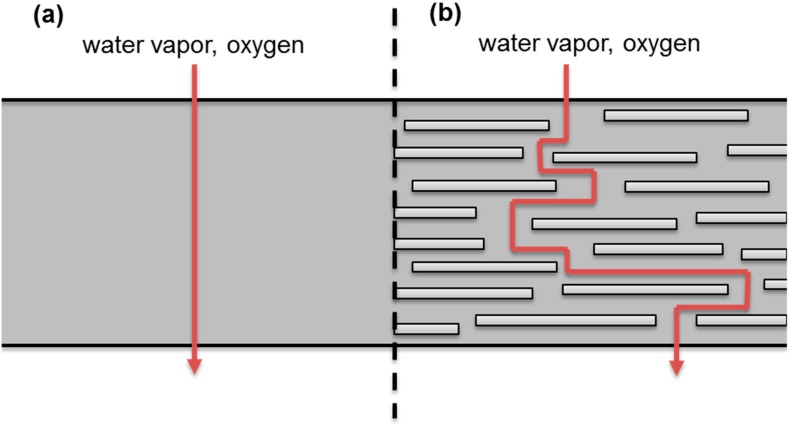
Illustration of the “tortuous pathway” created by the incorporation of exfoliated nanoplatelets into a polymer matrix film. Adapted from [23].

For example, it is well known that for the packaging application, the barrier improvements provided by nanocomposites depend on a so-called tortuosity effect where the nanofillers increase the gas diffusion pathway [22] (Figure 1).

Other effects, like the selective adsorption of gas molecules on the particles of a high specific surface area, are also involved. Nanoplatelets composed of clays or other silicate materials, such as montmorillonite [(Na,Ca)_0.33_(Al,Mg)_2_(Si_4_O_10_)(OH)_2_·nH_2_O)], a layered phyllosilicate composed of anisotropic layers separated by water molecules, are the most studied for this application. The platelets have a thickness in the nanometre range, lateral dimensions from tens of nanometres up to several microns, surface areas above 750 m^2^/g and an aspect ratio in the range of 100–500 [3].

**Figure 2:**
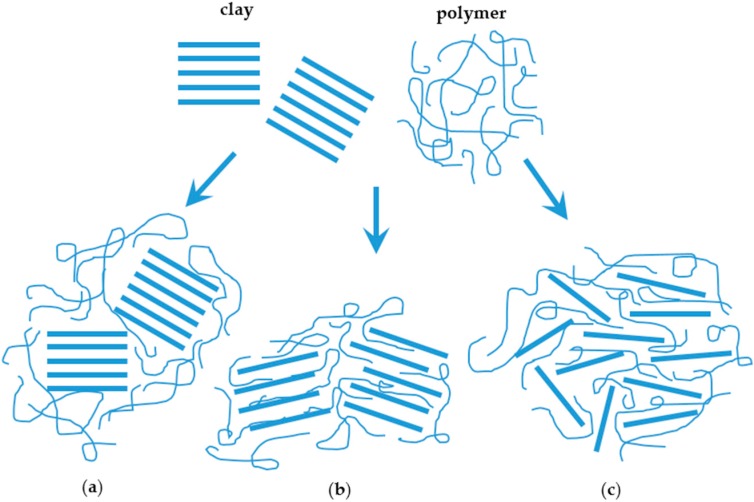
Scheme of different types of composite arising from the interaction of clays and polymers: (**a**) phase separated microcomposite; (**b**) intercalated nanocomposite; and (**c**) exfoliated nanocomposites. Adapted from [26].

When increasing the affinity between the matrix and fillers, nanoclay-based composites can show three different types of morphology: immiscible (e.g., microscale dispersion, tactoid), intercalated or exfoliated (miscible) composites [24,25] (Figure 2). The former of course does not allow the material to reach the properties promised by nanocomposites, while the latter two allow incremental improvements.

Several chemical or physical strategies have been used to improve nanoparticles’ dispersion (Table 1). Surface modifications by organosilanes or long chain alkyl ammonium ions can be used to enhance the compatibility of the matrix and fillers and improve the dispersion [27,28] or even in some cases covalently react them with the continuous phase. In terms of physical methods, besides the use of mechanical mixing methods (high speed/shear stirring in the case of wet processing like high energy ball milling [29], or extrusion in the case of thermoplastics, etc.), sonication can improve the nanoparticles’ dispersion both in solutions and melt polymers [30]. When applicable, in situ polymerization was also reported as a way to enhance dispersion rather than the addition of nanoparticles in media of higher viscosity [31,32]. The application of a high shear [33]/compression in one direction or, depending on the nature of the nanoparticles, of electromagnetic fields can also lead to the orientation of the nanoparticles and possibly maximize the tortuosity across a packaging, or increase the mechanical properties in a given direction. Likewise, the impact of dispersion on the flammability of a material is driven by the barrier to oxygen permeation that can prevent further feeding of combustion, as well as by a charring effect when nanoparticles form a cohesive surface protective layer to stop fire propagation [13,34].

Nanoclays are usually added at less than 10 wt % in the polymer matrix and in lower amounts than microfillers to achieve similar improvements in properties to those that microcomposites could present. When a good exfoliation of clays is reached, oxygen barrier improvement factors, typically in the range of two, but in cases reaching up to 15, as well as limited water vapour barrier improvements below two, can be found in the literature [16,35].

### 2.2. Conventional Approaches

Depending on the dimensional range needed and on the state of the material, several methods may have to be combined in order to get the full picture of the multi-scale morphology. X-ray diffraction (XRD), as well as scattering at different angles (small angle X-ray scattering (SAXS), wide angle X-ray scattering (WAXS), etc.), scanning electron microscopy (SEM), transmission electron microscopy (TEM), infrared spectroscopy (IR) or atomic force microscopy (AFM) are generally used to characterize the dispersion quality of nanoparticles in solid matrices [16]. The degree of intercalation or exfoliation for particles presenting a specific layered organization, such as clays, can be characterized by XRD [3], whereas small angle neutron scattering (SANS) can be used to characterize the fractal organization of amorphous particles, like fumedsilica [36]. TEM is commonly used to examine the dispersion of nanoparticles within a polymer and to determine qualitatively the morphological aspects, such as filler size, shape, distribution, etc.

There are three different types of composites arising from the interaction of clays and polymers: (a) phase-separated microcomposite; (b) intercalated nanocomposite; and (c) exfoliated nanocomposites (Figure 2) [26]. These three types of nanostructures that nanoclays can form when they are incorporated into polymer matrices (Figure 2) can be easily identified by XRD. The structure is considered a tactoid when the basal spacing of a mixture is the same as that of the clay cluster, with no polymer chains inside the clay gallery. In intercalated structures, the interlayer spacing is expanded (d-space increased); thus, the detector angle (2θ) position in the X-ray spectra is decreased. Fully-exfoliated structures show no peaks in XRD, indicating that polymer chains have penetrated the gallery and widened the interlayer space until the regular stacks of clay layers become disordered, so that the X-ray cannot detect any regular structure [3]. Protein-clay nanocomposite research by Chen and Zhang [37] demonstrated that montmorillonite tactoids were delaminated into thin lamellas in soy protein. The d-spacing values increased from 1.4 nm for the montmorillonite tactoid to a value ranging from 2–3 nm. Osman and co-workers also in a systematic way demonstrated by XRD that the length of the alkyl chains of ammonium salts incorporated into the inter-gallery space of modified nanoclays had an important influence on their degree of exfoliation and, as a consequence, in the gas barrier properties of layered silicate-Polyethylene (PE) composites [38]. As such, the gas permeation properties of polymeric nanocomposites films could also be used as an indirect technique to evaluate the dispersion of nanofillers [39]. However, gas permeation rate measurements usually take several hours and require complex systems that cannot be used for inline monitoring.

While the quantification is rather easy using the multiscale interlayer distances returned by XRD when the morphology of the nanocomposite allows it, it is more difficult to finely assess the dispersion quality when using TEM micrographs. This is due to the need to characterize many samples in order to obtain a statistically-relevant characterization and also due to the possible overlapping of several nanoparticles in the microtomed specimen seen in transmission. Consequently, some work has been reported using image analysis of TEM pictures in correlation with other techniques (e.g., XRD analysis of clays) [40] or alone when no other techniques were available [41]. This requires a quite complex image treatment to get a fine quantification of the multi-scale dispersion and distribution [42], and models, such as the Rosin–Rammler–Benett distribution function, have been used to determine the silica dispersion vs. ultrasonication time [12]. These disadvantages are also encountered for SEM cryofracture analysis, although this method is often used to assess nanostructure-property relations, especially for toughness [43].

Furthermore, melt rheology has been used to evaluate dispersion and microstructure in two Polypropylene (PP)/organo-clay (OC) nanocomposites prepared by melt blending. Very good correlation has been reported between the rheological properties and their dispersion state assessed by small angle X-ray scattering and TEM [44]. Rheology offered an integrated multi-scale picture of the composite material with reliability advantages compared to methods using smaller samples that are sensitive to micro-scale inhomogeneities. Likewise, such an approach was also qualitatively implemented using percolation models for fumed silica suspensions into epoxy prepolymers in the liquid state before polymerization [42].

Table 2 summarizes the main characterization methods to evaluate nanoparticles’ dispersion in solid matrices and the corresponding information provided.

The dispersion of nanoparticles in liquid media is usually evaluated by measuring particles’ size distribution with optical techniques, such as dynamic light scattering (DLS), nanoparticle tracking analysis (NTA) and laser diffraction. In the case of DLS, the Brownian motion of dispersed nanoparticles in a liquid causes laser light to be scattered at different intensities. Analysis of these intensity fluctuations yields the velocity of the Brownian motion and, hence, the particle size using the Stokes–Einstein relationship [45]. Similarly, for NTA, a laser beam is passed through a chamber containing the nanoparticles in suspension so that they scatter light in such a manner that they can be easily visualized with a microscope with a video camera. The NTA software tracks many particles individually and using the Stokes–Einstein equation calculates their hydrodynamic diameters [46]. Laser diffraction returns particle size distributions by measuring the angular variation in intensity of light scattered as a laser beam passes through a dispersed particulate sample. It is a widely-used particle sizing technique for materials ranging from hundreds of nm up to the millimetre size [47].

**Figure 3:**
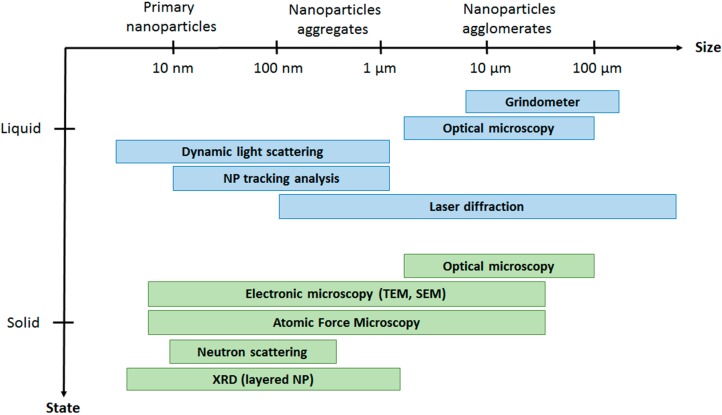
Conventional characterization techniques to evaluate nanoparticles’ dispersion in different media.

When the dispersion of nanoparticles in the different media is not efficient, nanoparticle agglomerates with sizes greater than 1–5 micrometres are usually formed. These micrometric agglomerates can be observed by optical microscopy or with specially dedicated devices, such as grindometers (in the case of liquid dispersions). Figure 3 summarizes the different conventional characterization techniques usually employed to evaluate the dispersion of nanoparticles in different media (solid or liquid state) depending on the particle size.

### 2.3. Inline Monitoring

As discussed above, the processing method and formulation can influence the dispersion of nanofillers in a polymer matrix and, therefore, its performance. In such a context, to allow a dynamic process/material optimization, there is a need to control the morphology of the nanocomposites in processing conditions. As mentioned, the most common methods for the post-process study of the morphology of polymeric nanocomposites are SEM, TEM and XRD; however, the implementation of these techniques inline is difficult and expensive. Previously-mentioned rheological trials [42,44] were carried out offline on samples acquired during melt processing. Nevertheless, equipping the extruder with an appropriate sampling system linked to a measuring chamber, it was also possible to run rheological trials inline during the melt processing [48], and it should therefore be possible to obtain in a short time some predictive parameters of the dispersion by using different morphological models. Besides, Bugnicourt et al. studied multiscale organization (particles, aggregates, agglomerate) covering ranges from nm to hundreds of microns all along the epoxy-silica nanocomposite process using several complementary techniques when the material changed from liquid (rheology, optical microscopy, dynamic light scattering, SANS) to the solid state finally (transmission electron microscopy, image analysis, SANS). Additionally, polymerization was investigated by confocal microscopy and SANS under a hot plate showing a competition between the structure of the fillers and the crosslinking of the matrix [42]. However, those methods, simulating the industrial process, are difficult to apply in real scale manufacturing.

Instead, several spectroscopic techniques have already been applied for inline monitoring of the extrusion process, such as ultrasound, Raman, UV-VIS (ultra-violet-visible) and NIR (near-infrared) spectroscopy, demonstrating that they were suitable non-invasive methods for process control without time consuming analysis and sampling, while inserting optical probes in the polymer stream [17,18,19,20]. This has often been coupled with other methods.

In particular, the use of NIR has been reported for parameters, such as moisture, composition in extruded fillers/polymers blends or reaction status in reactive extrusion [49,50,51,52]. Generally, in polymer processing, NIR is performed mostly in transmission mode [53] by using flow cell barrels between the extruder and die. The degree of exfoliation was determined offline as an indicator for the dispersion of the nanofillers in the polymer matrix (Polypropylene (PP) and Polyamide 6 (PA)) for different modified layered silicates and at different process conditions [54]. The use of reflectance mode by the comparison of inline diffuse reflectance and transmission measurements at the same location of the barrel of a twin screw extruder was also validated. To use NIR to predict the desired properties of the nanocomposites, here based on PP and montmorillonites, it was necessary to correlate data from the inline method with offline data through multivariate data analysis by chemometric analysis [55]. Successfully sampling the processed material without affecting its structure, e.g., altering the material morphology when using semi-crystalline polymers, is another issue to allow such monitoring. This was solved by a novel sampling device for extrusion that uses a vertical piston to eject the sample [56].

Likewise, inline oscillatory rheometry and inline NIR were used to study the evolution of dispersion during the preparation of a PP/PP-g-MA/organoclay system by twin screw extrusion [48], as confirmed by offline scanning transmitted electron microscopy (STEM), XRD and FT-IR analyses on samples collected from the extruder.

The use of high temperature ultrasonic sensors has also become more widespread for the monitoring of melt-processes of polymer blends and composites. As an indirect technique requiring calibration or artificial intelligence-based models instead of being properly a real-time monitoring technique, ultrasonic spectroscopy has been shown to be an efficient way to predict the dispersion of micro- and nano-fillers in the extruder as a function of process parameters [57,58,59], based on the monitoring of velocity and the attenuation of ultrasound in the flowing material. In the case of nanoclays in a low-density Polyethylene (LDPE) matrix, in-process ultrasonic measurements across melt flow in the extruder die were shown to provide a viable indication of dispersion levels, although the relationships between measured signals and morphology were complex and warranted further investigation [60]. The combination of NIR spectroscopy and ultrasonic measurements, which are in excellent agreement, was also reported as providing a highly reliable correspondence between the achieved process monitoring results and exfoliation level [54].

Nylon 6/clay nanocomposites were furthermore studied by dielectric relaxation spectroscopy (DRS) to correlate morphology and microstructure with the relaxation behaviour of the polymer matrix at the molecular level using an inline dielectric slit die sensor. Dielectric relaxation functions were affected by the presence of nanofillers depending on interfacial effects related to the dispersion, although rather qualitatively than quantitatively. Likewise, the measurement of electrical resistivity provides potential for inline dispersion monitoring in the case of conductive nanofillers, such as carbon nanotube nanocomposites processed by sonication in polyester resins [43].

As seen, most techniques tested for inline monitoring of nanoparticles dispersion are indirect and require calibration vs. offline techniques. Multivariate spectroscopic analysers (NIR, IR, Raman, ultrasounds, etc.), which seem the most promising to monitor the dispersion process in near real time, have been available commercially for several decades. The penetration of technology is still relatively low in the industry since process analytical chemistry (PAC) and the chemometrics behind are complex, highly dependent on the process and composition and not developed for all combinations. When it comes to monitoring the dispersion of nanofillers specifically, although some promising results have been published with nanoclay extrusion, extensive calibration campaigns are required to validate the use of the same approach for other nanoparticles and/or processes. Reported studies were generally carried out in simplified lab processes, but after an initial calibration, the combination of several of those techniques and predictive models has irrefutable potential for controlling the process so that a constant dispersion is reached in industrial processes working in fixed configurations and compositions.

## 3. Thickness Monitoring

### 3.1. Background

The thickness and consistency of nanocomposite coatings and nanodeposited layers (nanocoatings) have a high impact on their performance. On the other hand, depositing layers that are thicker than necessary is an inefficient use of materials, which can be very costly [61]. In addition, a homogeneous thickness distribution is a key for consistent product quality of the highest standard.

The reduction of gas permeability is inversely proportional to the barrier layer thickness applied [62,63], and the barrier effect can be further improved with the addition of small percentages of nanoparticles [24], so thin regions and holes in packaging barrier films for oxygen-sensitive products should be eliminated, as they can lead to the ingress of harmful oxygen and the spoiling of the stored product. Thin regions or holes in anti-wetting layers will lead to the accumulation of liquid and deposits, which can cause problems in the performance of the underlying structure, such as a solar cell.

Graphene, a monolayer of graphite, was also extensively studied recently. It improves a range of material properties, such as mechanical and electronic [64], but is also considered an ultrathin, perfect two-dimensional (2D) barrier against gas diffusion [65,66]. Instead of graphene monolayers, graphene nanoplatelets or graphene oxide (optionally reduced) are generally used in the materials development due to their lower cost of production on a large scale. Compared to other carbon-based nanoparticles, such as fullerenes or carbon nanotubes, graphene has a higher aspect ratio and therefore maximises the gas-diffusion pathway. As a further very positive perspective in terms of the use of graphene in packaging applications, graphene deposits were shown to have sufficient resilience to withstand thermoforming [67]. It was also demonstrated to extend the shelf life of beer packed in Polyethylene terephthalate (PET) bottles by a factor 2–5 [68]. In this study, diamond-like carbon (DLC) was deposited using a microwave plasma reactor to reach nanocoatings in the range of a 50-nm thickness leading to over a 10-fold decrease in the oxygen permeation. The optical properties of the coating were reported to vary from semi-transparent to fully transparent, depending on the technology used.

Other work reported the use of graphene to prepare superhydrophobic coatings. Nanostructured coatings in the micron thickness range were dipped, sprayed and brushed onto different adhesive substrates, such as metal, glass and paper. Due to the improved mechanical properties and good adhesion with the substrate, excellent retention of superhydrophobicity was observed even after scratching, therefore showing the potential for improving the long-term maintenance-free energy harvesting efficiency in the solar panel sector [69].

In applications where the nanocomposite has an active role, such as in the donor and acceptor layers of solar cells or as plasmonic structures for coupling light into active layers, the thickness is critical to the device electronic efficiency [70].

Uniform and accurate deposition or formation of nanocomposite and nanodeposited layers across large surface areas is challenging due to changes to the viscosity [71] and the tendency of the particles to agglomerate, as seen in the previous section [72]. Both the functional requirements for layer uniformity and the challenging processing conditions are motivating factors for closely monitoring the applied layer thickness and actively controlling the process. Additionally, on the other hand, there is the desire not to waste resources by over-engineering layers that are excessively thick, which could happen without accurate monitoring, but instead to ensure that they are thick enough for the required application.

### 3.2. Conventional Approaches

A wide variety of techniques have been used to measure the thickness of nanocomposite coatings and nanodeposited layers, from the high resolution and high cost, such as cross-section scanning electron microscopy (SEM) [15,73,74,75] and cross-section transmission electron microscopy (TEM) [14,15], to lower cost and lower resolution techniques, such as profilometry [76,77] and cross-section optical microscopy [71]. SEM and TEM are widely used in research because of their high resolution, to the nanometre level and below, whereas profilometry and optical microscopy are more often used in industry, because they are more economical and simpler to implement. Other important factors are the range required; for example, optical microscopy is not practical for sub-micrometre films, and the resolution is limited to approximately 300 nm in air. The resolution of profilometry is nanoscale [78], but the accuracy of techniques can also be affected by sample preparation; so for example, in cutting cross-sections for optical microscopy with a blade, care must be taken not to deform the layers; while film thickness measurement with profilometry relies on making a scratch that cleanly removes the film down to the substrate to produce a step that is the height of the layer, and it is not always possible to ensure that this happens.

However, all of these techniques are only applicable by first sampling the material and bringing the samples to the metrology instrument, often in a specialised laboratory or cleanroom. In the case of TEM and SEM, sample preparation can take up to several hours. For TEM, samples must be encapsulated in resin and then microtomed to slices that are several hundred nanometres thick [79], while for SEM, samples often need to be cryogenically frozen before cleaving to make a cross-section surface with clearly-defined layers, and then, they can require the deposition of a conductive metal coating [80]. Although less preparation is required in the case of optical cross-section microscopy, still the sample must be carefully mounted between microscope slides. This is time consuming and means that only a small portion of the product can be tested. These techniques require that a physical sample be cut from the fabricated product, thereby damaging the sample piece, so they are invasive and destructive. For industrial processes involving such nanocoatings, for example as a stage in a laminate production, often performed in roll-to-roll configuration, it is only practical to take a physical sample at the end from the final product, rather than during the manufacturing process, which leads to the possibility that a serious defect occurring early in the process can lead to abandoning the product only after subsequent, costly, processing steps have been completed.

### 3.3. Inline Monitoring

As has been seen, TEM, SEM, profilometry and generally all of the techniques previously listed to measure thin coating thickness can only be used offline. In monitoring a manufacturing process, the most useful configuration is for the metrology instrument to be in line with the process [81]. In this way, the process can be monitored at all times, and the greatest sampling of the material can be achieved, up to and including all of the surface area. Analysis can be performed in a short time interval, so that process deviations can be quickly identified and corrected. Inline process monitoring has been adopted by the semiconductor and the pharmaceutical industries [82,83].

The most common inline or in situ process monitoring technique that is used for nanoparticle or nanolayer deposition is a quartz crystal microbalance, which is used for a range of processes, such as evaporation, sputtering or electrodeposition in solution [15,84]. In this case, the parameter measured is mass deposited per centimetre squared. However, this technique is not suitable for monitoring a roll-to-roll process for a long duration, as the quartz microbalance continuously accumulates the deposited material that is spread on the moving film and so becomes completely covered and exhausted in a relatively short space of time.

More suitable techniques for inline integration are optical, whose benefits include being accurate, non-destructive, non-contact and requiring little or no sample preparation. However, while inline optical metrology is used in a wide range of industries to monitor homogenous, monolithic film thickness in deposition processes, such as printing and lamination, little work has been done on a dedicated solution for inline monitoring of nanocomposite and nanodeposited films, due to the increased complexity of the optical signal. As discussed below, there has been a good deal of research activity undertaken in academia to characterize the thickness and optical properties of nanocomposites using optical techniques, mainly with effective medium approximations to describe the dielectric function, but there has been little development towards applying these results to inline monitoring solutions for industrial processes.

The range of optical metrology techniques is large and in relation to the current topic, they can be thought of as falling into two groups: on the one hand those currently used for process monitoring of homogeneous thin films and, on the other hand, those used for offline thickness measurement of nanocomposite films, largely in the research community.

The simplest industrial inline techniques are variations of machine vision, in which the optical density (relative brightness) or colour of the sample is calibrated against samples of known thickness. Versions of such relative thickness measurement systems are the most commonly used commercially on moving film deposition processes, with typical examples being provided by Dr. Schwab Inspection Technology GmbH [85] and Dr. Schenk GmbH [86] for roll-to-roll printing of films for organic electronics and packaging barriers, and also structured surfaces, such as optical storage discs, solar panels and microfluidic devices. Relating colour to thickness suffers from the problem of repetition, since the same colours are generated from films whose thicknesses vary by approximately a wavelength of light. Recent work by Dr. Schenk in the EU-funded project Clean4Yield (Clean4Yield (Contamination and defect control for increased yield for large scale roll-to-roll (R2R) production of organic photovoltaic (OPV) and OLED), Project Number FP7-CP-IP 281027) has led to a four-colour system, in which the different sinusoidal variations in brightness of each of the four colours is monitored individually. Since each colour varies at a different rate, repetitions in the combined signal are avoided, and the range over which a unique thickness measurement value can be ascribed is increased (Clean4Yield project [87] and the video [88]). A relative accuracy of 2% of the layer thickness was achieved, which for 2–3 µm-thick films is approximately 40–60 nm [87].

These imaging, relative thickness, techniques have the benefit of being combined with conventional machine vision to relate thicknesses to features in the film, either designed structures or accidental defects. However, the main drawback is the need to calibrate against an absolute measurement, and for example, the Clean4Yield demonstration system used inline point ellipsometry measurements, provided by a Horiba system (see the discussion below) to calibrate the relative thicknesses obtained with Dr. Schenk’s imaging system.

Optical absorption spectroscopy has been used extensively in research since it provides both thickness and chemical structure information, which is more relevant at the development stage of new processes, to monitor materials’ growth and assembly processes. As with the imaging techniques mentioned already, thickness values must be assigned by calibrating against a quantitative measurement technique, usually performed offline, such as cross-section SEM, profilometry or ellipsometry. UV-VIS spectrometry has been used to characterize the layer-by-layer growth of bilayers of Polyamidoamine (PAMAM) dendrimer and Poly(styrene sulfonate) (PSS) or Poly(acrylic acid) (PAA) with Ag nanoparticles [77]. Absorbance at individual wavelengths related to structural features was used in this case, such as Ag plasmons and phenyl rings. Precision was defined in terms of the numbers of layers, with thickness of hundreds of nanometres. VIS-NIR spectrometry on samples of dense films of montmorillonite/Poly(vinyl alcohol) could distinguish samples in steps of 25 layers in the deposition process, or 125 nm [72]. Spectroscopic techniques have largely been used in offline mode to date. However, FT-IR spectroscopy has been used in situ for deposition processes to monitor the chemistry of layer growth on a fixed substrate [89].

To measure the thickness of layers by the optical technique without a comparison to known samples, it is necessary to measure the changes in either the spectrum or the polarization state of the light, which is caused by the interaction with the top and bottom boundaries of the thin layer, as well as the thickness. Once the fundamental optical properties of the materials being studied can be established, such as the complex refractive index, the interaction of the light with the layer can be modelled, and the thickness of the layer can be calculated. These techniques fall under the headings of interferometry, spectral reflectance and ellipsometry. In general, interferometry or spectral reflectance are much simpler and less expensive than ellipsometry, but are restricted to measuring less complex structures.

Low-coherence interferometry, which is also known as optical profilometry, makes use of the bright fringes of constructive interference that occur when low-coherence light, such as from a super-luminescent LED, which has been reflected from the sample surface, is combined with light reflected from a reference optical path of exactly the same distance. The distance to the top surface of the sample and to all of its internal interfaces is found by scanning the reference path length [90]. This technique is very effective at distinguishing the reflections from multiple layers in a stack and can measure distances to a precision of 0.1 µm. Commercial versions are used for inline monitoring of industrial processes (Lumetrics Inc., Eagle’s Landing Business Park, 1565 Jefferson Road 420, Rochester, NY 14623, USA; Novacam Technologies Inc., 277 Lakeshore Road, Suite 04, Pointe Claire, QC H9S 4L2, Canada), but this requires a minimum layer thickness of approximately 12 µm, or greater than the coherence length of the light source. For this reason, it is used to measure micro- to macro-scale layer thicknesses, from 12 µm–25 mm, including the walls of medical devices and tubes, contact lenses, touchscreen display multi-layer stacks [91], small mechanical parts and food packaging materials [61].

Spectral reflectance or reflectometry is the most widely-used technique in industry that provides absolute, quantitative data without the need to calibrate against samples of known thickness, for sub-micrometre samples. A white light source is directed at the sample surface, and the reflected light is collected and analysed with a spectrometer. Thickness is calculated by identifying the wavelengths of the interference peaks in the reflectance spectrum, where the thickness of the layer is a function of the peak wavelength and refractive index of the material [92,93]. The greatest accuracy is obtained when more than one peak can be identified and clearly resolved, so the technique is ideally suited for the thickness range of 1–50 µm, but is commonly used for thicknesses of 100 nm–1 µm, and in a well-controlled laboratory setting, with very smooth substrates, a minimum thicknesses of 5 nm can be measured with nanometre precision (SENTECH Instruments GmbH, Schwarzschildstrasse 2, 12489 Berlin, Germany). Spectral reflectance can be deployed via fibre optic cables, and use can be made of this to measure an array of points across a roll-to-roll web (Filmetrics Inc., 10655 Roselle St., Ste. 200, San Diego, CA, USA; Dr. Schwab Inspection Technology GmbH, Industriestrasse 9, 86551 Aichach, Germany). Application areas include homogeneous films for microelectronics, touchscreens, OLEDs, ophthalmic coatings, solar cells, anti-reflection coatings and optical discs. For inline applications, fast analysis is essential to obtain location-specific information. Recent work by Horiba has achieved acquisition times of 1–10 ms, with NIR reflectometry for thick films of several 10 s of micrometres [94].

Recent work in the EU Framework 7 project THIME [95] has led to the development of an imaging spectral reflectance system, validated as an inline quality control system for monitoring thin film thickness in roll-to-roll organic photovoltaic (OPV) production processes. The system makes use of a hyperspectral imaging camera to obtain spectral data from a line of several hundred pixels across the web width and can perform analysis from all points in parallel to calculate a complete thickness map of the printed web. It has been used on dried layers of PEDOT on PET and P3HT:PCBM on PET, for thickness ranges of 150–600 nm, achieving an accuracy of approximately 15–30 nm, varying with thickness range.

Table 3 summarizes the different techniques that have been used offline and inline for the characterization of nanostructured coatings and nanolayers.

Industrially, spectral reflectance has been used to characterize complex optoelectronic inks, but it has been studied offline to measure the thickness and refractive index of true nanocomposites and nanodeposited layers. Al/Al_2_O_3_ films grown by thermionic vacuum arc [96] on glass substrates, with a thickness of 60 nm, have been measured, and colloidal structures of silica particles in a polymer matrix, achieving an uncertainty of ±5% of overall thickness for films in the range of tens of micrometres [97]. For the latter material, the effective refractive index is measured using a prism-coupled refractive index measurement system from Metricon (Metricon Corporation, 12 North Main Street, Pennington, NJ 08534, USA), and this value is used for thickness measurements. The effective refractive index is also calculated using an effective medium approximation (EMA), where the combined refractive index is a weighted sum of the indices of the components of the medium, and this agrees well with the measured value. The effective medium approximation [98] can be used for composite layers in which the structure size, like particles, bubbles or voids, is smaller than the diffraction limit of the incident light, so the particles do not interact with the light individually. This sets a maximum dimension limit of approximately several hundred nanometres for visible light, depending on the morphology of the structures, so it is well-suited to nanocomposites.

Ellipsometry measures a change in polarization as light is reflected from a surface. When linearly-polarized light is reflected from a surface, in general, the amplitudes of its two perpendicular electric field components change, and so does the phase between them, and the light becomes elliptically polarized. The polarization change is represented as an amplitude ratio and a phase difference. The measured response depends on the optical properties and thickness of individual materials, and therefore, ellipsometry is primarily used to determine film thickness and optical constants [99]. By measuring reflectance at non-normal incidence, ellipsometry is more sensitive to very thin layers, of Angstrom thickness. For example, it has been used to measure the thickness of single-layer graphene, with a water layer on top, each of which have thicknesses of 1–5 Å [100]. This result was achieved with an Accurion imaging ellipsometer (Accurion GmbH, Stresemannstr. 30, 37079 Goettingen, Germany), which provides thickness maps at the micron scale. The trade-off with ellipsometry in general compared to spectral reflectance is a more complex and costly system, as well as a slower speed of operation.

The same researchers have performed ellipsometry in situ to study the evolution of processes on fixed substrates, such as the drying of deposited very thin graphene films [101], using a Sopra GES5E-IRSE (Sopra SAS, Avenue de l’Europe, 92400, Courbevoie, France). Similar ellipsometers with a reaction chamber for in situ monitoring of surface processes on the laboratory scale are manufactured by Sentech (SE 400adv) (SENTECH Instruments GmbH, Berlin, Germany) and Horiba (Uvisel In-Situ) (HORIBA UK Limited, 2 Dalston Gardens, Stanmore Middlesex HA7 1BQ, UK). Ellipsometers with automated panel or wafer loading are available for semiconductor or flat panel display manufacturing process lines, for example from Semilab (SE-3000 (Semilab Semiconductor Physics Laboratory Co. Ltd., Prielle Kornélia str. 2. H-1117 Budapest, Hungary) and FPT-6 (Semilab Semiconductor Physics Laboratory Co. Ltd., Prielle Kornélia str. 2. H-1117 Budapest, Hungary) or Horiba (HORIBA UK Limited, 2 Dalston Gardens, Stanmore Middlesex HA7 1BQ, UK) or Sentech (SENDURO) (SENTECH Instruments GmbH, Schwarzschildstrasse 2, 12489 Berlin, Germany). These are best described as at-line systems, as they comprise a separate, modular step in the manufacturing process, rather than being integrated with existing processing machines.

For the reason of the complexity of the technique, effective inline ellipsometry systems for monitoring moving or roll-to-roll fabrication processes are few. A system has been developed with single point monitoring, which can scan the sample surface with movement stages [102,103]. This has been used to monitor roll-to-roll printed organic solar cell layers on a lab-scale system.

A commercial inline system has been developed by Horiba (Uvisel Inline) (HORIBA UK Limited, 2 Dalston Gardens, Stanmore Middlesex HA7 1BQ, UK). This system has been used to characterize barrier films of homogeneous metal layers on PET and tested at an industrial site [104], using the methodology of Logothetidis mentioned above [102]. Recent work in the Clean4Yield project has led to improved acquisition times of 1–10 ms and the addition of an index-matching roller to reduce the strength of back-reflections from the underside of the substrate [87].

An inline imaging ellipsometry solution has been developed in the THIME project for roll-to-roll printing [95], which is capable of measuring thickness across a 30-cm width. For the first time, this enables thickness monitoring across the full area of a coated web, generating complete contour images of layer thickness. The technique makes use of very fast polarization switching rates with a laser line source.

Ellipsometry has been used extensively in offline mode in a research setting to measure the thicknesses of thin nanocomposites, with thicknesses of less than one micrometre, such as clay platelets in polyvinyl alcohol [72] and luminescent layers of Eu:GdVO4 nanoparticles covered with a silica protective coating [105].

Ellipsometry has been performed in situ for fixed deposition processes, at the laboratory scale, for example of CdTe on Au by electrochemical deposition [106]. Again, an effective medium approximation is used to calculate the dielectric function. Process monitoring of nanocomposites and nanodeposits by ellipsometry in industrial production has not been reported, with possibly the greatest challenge being the application of the more complex effective medium modelling within the fast sampling times required of continuous production processes.

## 4. Conclusions

In spite of the huge progress made in the field of nanocomposites during the last few decades, a full control over their morphology (nanostructure dispersion or even orientation) is still desired to consistently reach optimal properties. Same goes for nanocoatings, as well as nanostructured coatings, the performances of which are highly influenced by their thickness and homogeneity.

This paper reviewed some of the key properties that are influenced by dispersion and thickness, as well as the way these parameters are generally measured in the literature by offline methods, such as, respectively, XRD, TEM and profilometry. As briefly illustrated in the background for this article, the achievement of an optimal nanoparticle dispersion in bulk nanocomposites, or a constant nanocoating, or nanocomposite coating thickness, offers great potential in terms of antimicrobial, gas barrier, water vapour barrier, water repellence or flame retardant surface functionalization and interesting prospects for solar panels, packaging and automotive fields. Required mechanical reinforcement and flame retardance for automotive applications can both benefit from the optimal dispersion, whereas nanocoatings can also improve the latter property. In terms of solar energy, surface nanocoatings can help with achieving self-cleaning panels, but also tune the optical properties, for example screening the unnecessary UV light or increasinge the gas/humidity barrier.

Various optical technologies have shown promising results for the inline monitoring of polymer nanocomposites in terms of nanoparticles dispersion (especially NIR and Raman spectroscopy with the potential for implementation both in liquid media and melt plastic streams) and nanocoatings’ thickness (e.g., spectral reflectance and ellipsometry-based systems). Nevertheless, all of these results, which could ease the manufacturing of nanocomposites, still have to be channelled to the industry. Indeed, due to the number of parameters involved, unless an effective monitoring system is used, the measurement of dispersion can be unreproducible or the coatings may have to be thicker than needed to compensate for possible defaults and irregularities. This has led the industry to lose confidence in nanocomposites, due to time, cost and resource inefficiency, both from the design (over-engineering) and waste (scrapped parts which do not meet specifications) points of view. Together with process optimization, the future inline monitoring capabilities are expected to boost the field of nanocomposites. Having access to nanoparticle dispersions, but also to nanocomposite and nanocoating thickness monitoring systems (the latter being the object of current developments within the EU-funded project OptiNanoPro [107]) will help with running faster process optimization in order to reach suitable dispersion and also to guarantee constant quality, pre-requisites to achieve the cost-effective production of nano-enabled materials and devices in the industry.

## Figures and Tables

**Table 1:** Strategies for nanoparticles’ dispersion.

Chemical Dispersion Strategies	Physical Dispersion Strategies
Surface modification of nanoparticles - to enhance matrix/nanoparticles compatibility - to covalently react them with the continuous phase	Mechanical mixing by: - application of high shear - high energy ball milling - extrusion
	Sonication
In situ polymerisation	Orientation by: - application of compression/shear in one direction - electromagnetic fields

**Table 2:** List of conventional approaches to evaluate the dispersion of nanoparticles in solid matrices and the type of information provided.

Characterization Technique	Type	Information Provided
XRD	Direct	Exfoliation degree of layered nanomaterials
TEM	Direct	Filler size, shape and distribution
SEM	Direct	Filler size, shape and distribution
AFM	Direct	Filler size, shape and distribution
SANS	Direct	Fractal organization of amorphous particles
Melt Rheology	Indirect	Degree of dispersion
Gas permeation	Indirect	Degree of dispersion

**Table 3:** Thin film thickness measurement techniques.

	Offline Technique	In Situ or Inline Technique
Absolute thickness	- Profilometry	- Low coherence interferometry
	- Cross-section SEM	- Ellipsometry
	- Cross-section TEM	- Spectral reflectance
Relative thickness		- Quartz microbalance
		- Machine vision based
		- Optical absorption spectroscopy
